# Platelets Contribute to BBB Disruption Induced by HIV and Alcohol

**DOI:** 10.4172/2329-6488.1000182

**Published:** 2015-01-19

**Authors:** Madhavan Nair, Jose MB Maria, Marisela Agudelo, Adriana Yndart, Mayra E Vargas-Rivera

**Affiliations:** 1 Professor and Chair, Institute of Neuro-Immune Pharmacology, Department of Immunology, Florida International University, Miami, FL, USA; 2 Professor, School of Integrated Health and Science, Department of Art and Science, Florida International University, Miami, FL, USA; 3 Institute of Neuro-Immune Pharmacology, Department of Immunology, Florida International University, Miami, FL, USA; 4 School of Integrated Science and Humanity, College of Arts and Sciences, Florida International University, Miami, FL, USA

**Keywords:** HIV, Platelets, Thrombocytopenia, Alcohol, Blood Brain Barrier, Neuro-Inflammation

## Abstract

The role of platelets in the neurological diseases that underlie cognitive impairment has attracted increasing attention in recent years. Multiple pathways in platelets contribute to host defenses, as well as to CNS function. In the current study, we hypothesize that the Blood Brain Barrier (BBB) is disrupted when exposed to platelets from patients with triple Co-morbidity (hazardous alcohol users+ HIV+ thrombocytopenia), compared to those with dual, single or no morbidity (HIV only, alcohol only or healthy controls).

## Introduction

Thrombocytopenia (TCP: < 150,000 per microliter a 2.5^th^ lower percentile of the normal platelet count distribution) is a hematological disorder that affects 15% of the early HIV stage cases, and one-third of those with acquired immunodeficiency syndrome [1]. The mechanisms leading to TCP among people living with HIV are multiple, and include, but are not limited, to: HIV-associated bone marrow alterations, suppressed mega-karyocytopoiesis, immune-mediated platelet destruction, and oxidative stress [2,3]. Many of the drugs used in the clinical management of HIV have also been associated with thrombocytopenia, including antibiotics (i.e., trimethoprim-sulfmethoxazole, clarithromycin), anti-fungals (i.e., pentamidine, fluconazole), antiviral treatments (i.e., ganciclovir, alpha-interferon), and some antiretroviral drugs [4]. However, despite of ART, TCP persists in a subset of subjects, and recur with treatment interruptions [5–7]. Hazardous use of alcohol, which is highly prevalent among people living with HIV, could be another contributor [8]. Overall in the general population, TCP may affect 3–43 % of non-acutely ill, and 14–81 % of acutely ill, hospitalized alcoholics [9]. Furthermore, another plausible explanation of TCP is a shortened platelet lifespan, associated with platelet activation and subsequent clearance by the immune system [10].

Although thrombocytopenia rates have been reduced with ART, this condition is still relevant given its consistently found association with HIV disease progression [8,11–13]. Platelet count has been correlated with HIV viral load in both human and animal models [11–16]. More and more evidences still indicate that thrombocytopenia is a strong prognostic marker of death in PLWH. For example, in the Women Integrity Study, women having a platelet count of <50,000 cells/mm^3^ are at more than five-fold increased risk of dying due to any cause, and at three-fold increased risk of death due to AIDS, compared to those with normal platelet counts [17]. These multiple reports correlating platelet counts and CD_4_, viral load, disease progression and mortality, suggest that platelets play a critical role in the pathogenesis of HIV infection.

In addition, advances in platelet biology have uncovered new roles for platelets beyond hemostasis. In this regard, our group demonstrated that TCP is strongly associated with both, mood and cognitive impairments associated with HIV [8]. This finding was not unexpected, given that platelets are a source of two key neuro-immune factors: serotonin and Brain Derived Neurotropic Factor (BDNF). Similarly, Watchman et al. [18] identified a close relationship between platelet’s decline and increased risk of HIV Dementia. Individuals exhibiting a platelet decline of 100,000/µL or more were twice as likely to develop dementia. This risk was independent of virologic control, antiretroviral therapy, concurrent HIV-related illness, duration of infection, and levels of education [18]. Yet the exact mechanisms mediating these observations need to be identified if corrective measurements are going to be implemented.

At the early stage, HIV-1 enters the brain through an intact BBB, using Blood-Brain Barrier (BBB) efflux transporters [19]. At later stages, the Blood-Brain Barrier is disrupted, allowing the entrance of infected immune cells [20]. The relevance of the BBB in the HIV Neuro-pathogenesis associated neurological disorders was demonstrated early in the epidemic, when HIV/SIV was directly inoculated into the brain [21,22]. Though scientists expected that CNS damage would happen with increased frequency, quite the opposite was found, highlighting the importance of BBB disruption in the neuropathology of HIV [22]. Furthermore, observations that brain zones, such as the area postrema, that are not near the BBB, rarely exhibited HIV related lesions provided further support for this postulate [23]. The precise mechanism by which the integrity of the BBB is disrupted is still under investigation. In addition, since ART has failed to control BBB leakage [24,25], the need of additional studies has become even more apparent. Several hypotheses have been raised to explain the pathophysiology of BBB disruption in HIV; however, none of them by itself can explain the pathogenesis. The most accepted theory is immunological, and considers that BBB disruption can be explained by an excessive release of pro-inflammatory cytokines [19]. Noteworthy, investigations with other infectious diseases have demonstrated that platelet alterations can lead to aberrant Blood - Brain Barrier (BBB) permeability [26]. For example, thrombocytopenia has been observed during cerebral malaria [27]. During dengue infection, TCP has been recognized as a risk factor for the development of neurological symptoms [28].

Notably, platelets are the source of multiple pro-inflammatory substances, including TNF and IL-6, which have been associated with BBB disruptions [29,30]. They are also elevated in the circulation of PLWH that have HIV-associated neurological disorders [31,32]. In addition to pro-inflammatory cytokines, platelets are the source of the inflammatory mediator soluble CD40L (sCD40L), which recently was associated with BBB dysruptions [33]. Indeed, it has been estimated that platelets produce up to 95 % of all sCD40L found in circulation [33]. These findings led us hypothesize that TCP and the increase platelet activation promote an inflammatory response that may increase endothelial apoptosis leading to the BBB disruption.

Although the relationship between HAU and cognitive impairment has been studied, the role of alcohol in disrupting the BBB has rendered different results. For example, studies by two different teams of researchers have demonstrated that alcohol in conjunction with gp120 or Tat, promotes neurotoxicity [34,35]. On the other hand, Collins and colleagues [36] found that moderate amounts of alcohol can offer protection against gp120-mediated neurotoxicity. Findings were later confirmed by Belmadani’s team [37]. Determining if hazardous alcohol use impairs the BBB is worth of investigation given the wide prevalence of alcohol among people living with HIV [38].

Given the lack of data regarding platelets effects on the BBB, and the lack of conclusive data on alcohol’s role, we decided to fill these gaps. Specifically, we posit that the Blood Brain Barrier (BBB) will be more likely to be disrupted when exposed to platelets from patients with triple co-morbidity (Hazardous Alcohol Users + HIV+ thrombocytopenia), compared to those with dual, single or no morbidity (HIV only, alcohol only or healthy controls).

## Methods

We evaluated the effect of HIV, alcohol and thrombocytopenia on the BBB integrity using an *in-vitro* BBB model constructed with primary human brain micro vascular Endothelial Cells and astrocytes. The BBB membrane integrity was measured by Trans Endothelial Electrical Resistance (TEER). Para cellular permeability was established using Fluorescein Isothiocyanate (FITC)-dextran.

### Study population

Four hundred participants were enrolled in the parent study (The Platelets Mediating Alcohol and HIV Damage Study (PADS). PADS is a large, single-site multi-ethnic cohort, consisting of 400 people living with HIV (PLWH), of which 200 are Hazardous Alcohol Users and 200 are non-hazardous alcohol users. Non-ambulatory patients, and those presenting with major medical co-morbidities, such as CNS opportunistic infection, head injury, tumors, major psychiatric disease, developmental disorders, severe malnutrition, chronic renal failure, intestinal pathology, thyroid problems, cardiovascular or immune-based disease (i.e., malignancies, autoimmune diseases, or arthritis) were excluded. In addition, based on medical records, participants who had cirrhosis or active viral hepatitis were not eligible. Otherwise, the subject was enrolled. Five HIV negative subjects (3 alcohol users=HNAU, and two control subjects, HIV (−) / HAU (−), and 8 HIV positive individuals were age and gender matched. HIV positive were selected for being ART treated, with no past or present history of major comorbidities and no drug. Subjects were selected if their CD4 was between 200–350 cell counts, to assure that the participants were neither a fast nor a non- HIV-progressors. The HIV infected group included alcohol users (“HPAU”, n=5), HIV (+) non-alcohol users (“HPNA”, n=3), were recruited for this study.

### Ethics statement

Both, PADS and the pilot study, were approved by the central governing Institutional Review Boards at Florida International University and University of Miami. The study was conducted according to the principles expressed in the Declaration of Helsinki. Those participants who provided written informed consent and a signed medical release form were enrolled.

### Platelets were isolated from plasma human samples

Blood was collected by venipuncture into plastic tubes containing EDTA as anticoagulant. Whole blood from the participants was centrifuged at 2503 g for 15 min, at 22°C, and gently re-suspended in PBS for further analysis. Platelets were counted using a hemacytometer. This method has shown to render a purity of isolated platelets of 99%.

### Cell culture

Primary cultures of human brain micro vascular Endothelial Cells (HBMECs; catalog no. 1000) and human astrocytes (HAs; catalog no. 1800) were purchased from Sciencell Laboratories (Carlsbad, CA) and cultured as per supplier’s instructions. Primary HBMECs and HAs were obtained from above discussion.

### The Blood Brain Barrier *in-vitro* model

The BBB model was established according to the procedure described earlier [39]. The model consisted of two-compartment wells in a culture plate, with the upper compartment separated from the lower by a cyclopore polyethylene terephthalate membrane (Collaborative Biochemical Products, Becton Dickinson, San Jose, CA) with a pore diameter of 3 µm. In a 24-well cell culture insert, 2 × 10^5^ primary HBMECs were grown to confluency on the upper side whereas a confluent layer of primary HAs (2 × 10^5^ cells/insert) was grown on the underside. Intactness of the BBB was determined by measuring the Trans Endothelial Electrical Resistance (TEER) using Millicell ERS microelectrodes (Millipore, Billerica, MA). The electrical resistance of blank inserts with medium alone was subtracted from TEER readings obtained from inserts with confluent monolayers. The resulting TEER values represent the resistance of the endothelial cell monolayers. The BBB model was used for experiments at least 5 days after cell seeding. The BBB constructs were treated with 1 × 10^6^ platelets obtained from human blood donors categorized as HIV(+) Hazardous Alcohol Users (HPAU) with thrombocytopenia and without thrombocytopenia, HIV (+) non-Hazardous Alcohol Users (HPNA), HIV (−) alcohol users (HNAU) and normal subjects (CT). TEER measurements were performed at 48 h after adding the platelets. The results are presented as percent of control. As shown in the Graphical Representation


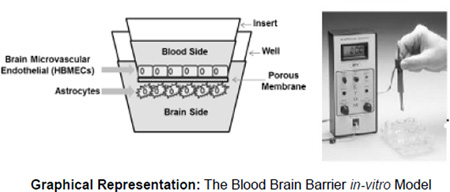


### FITC-dextran transport

The effect of platelets on the integrity of the *in vitro* BBB model and paracellular transport of flourescein isothiocyanate–labeled dextran (FITC-dextran; molecular weight 40000) were measured according to the procedure described earlier [20]. After the integrity of BBB was established by TEER measurement, the BBB monolayers were treated with 1 × 10^6^ platelets from Human samples and incubated for 48 h. After incubation, 100 mg/ml FITC-dextran (Sigma Aldrich, St. Louis, MO) was added to the upper chamber of the inserts and further incubated for 4 h. Samples were collected from the bottom chamber after 4 h and fluorescence intensity was measured at excitation wavelength 485 nm and emission wavelength 520 nm using Biotech Synergy HT multimode microplate reader instrument. FITC-dextran transport was expressed as percentage of FITC-dextran transported across the BBB into the lower compartment compared to untreated control cultures.

### Statistical analyses

All the data were analyzed using Graph Pad Prism software. Comparisons between groups were performed using one-way ANOVA and Dunn’s Multiple Comparison Post Test. Data is expressed as mean percent of controls. A Bonferroni value of p<0.05 was considered significant.

## Results

### Study population

All the HIV infected participants were receiving antiretroviral therapy at the time of blood draw. None of them had an active infection at the time of enrollment, and neither of them had other viral co-infections (i.e. viral hepatitis).

### The BBB is disrupted by platelets from HIV infected patient and/or alcohol users

**Figure 1:**
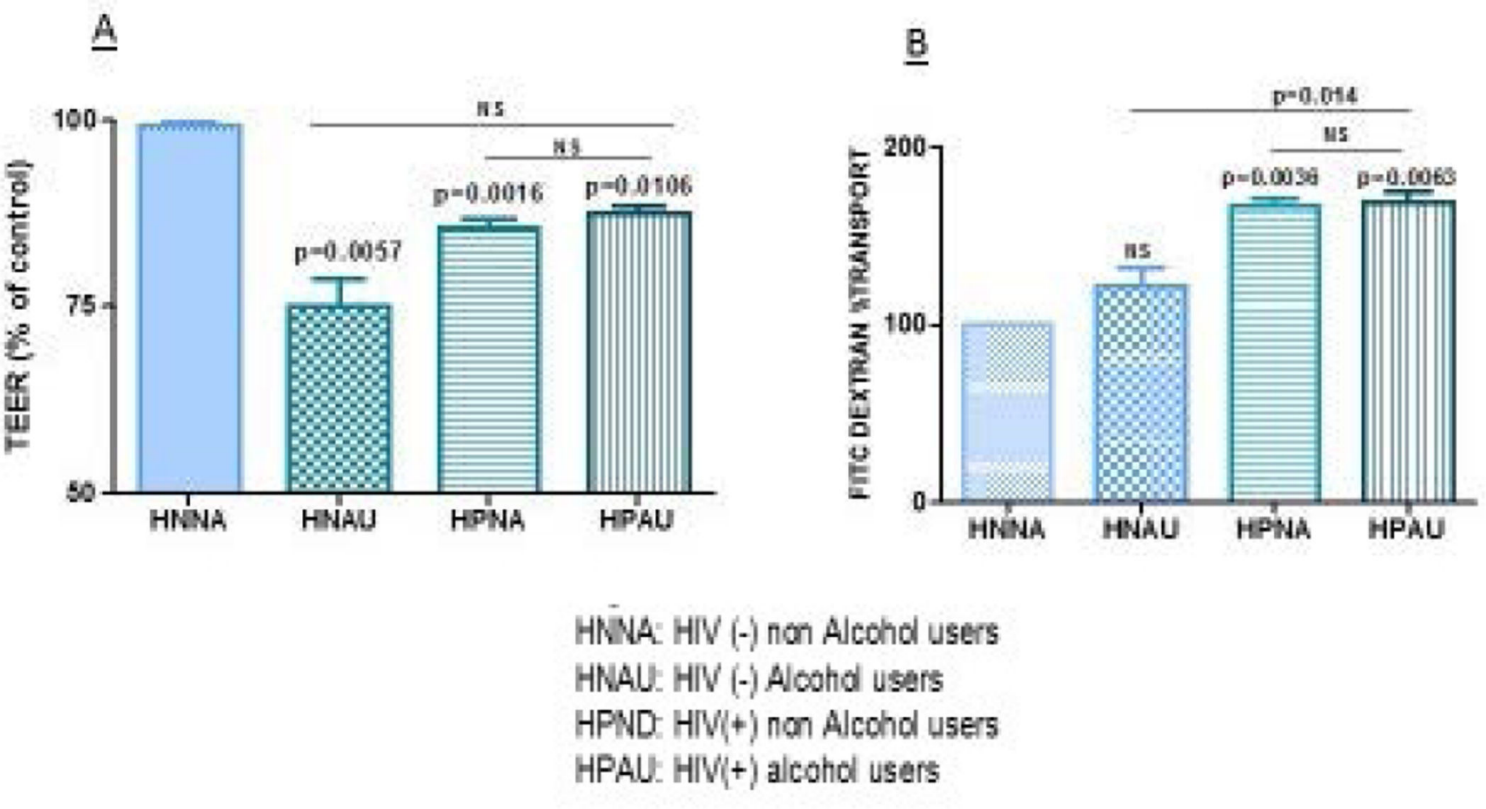
**a, 1b:** BBB is disrupted by platelets from HIV infected patient and/or alcohol users BBB disruption was observed when the BBB was treated with platelets from HIV negative alcohol users (HNAU), HIV positive non-alcohol users (HPNA), and HIV positive alcohol users (HPAU) compare to the HIV negative non-alcohol user controls The BBB permeability was significantly affected when the BBB was treated with platelets from HIV positive patients (HPNA) and HIV positive alcohol users (HPAU) compare to the controls. There was also an increased in permeability in the BBB treated with platelets from HNAU compare to Controls; however, it was not significant (CONTROLS = 100 vs HNAU = 75.19 *p* = .0057; CONTROLS =100 vs HPNA = 8.6 *p* = .016; CONTROLS =100 vs HPAU=87.48 *p* = .0106). The BBB treated with platelets from HPAU did not show any significant differences compare to BBB treated with platelets from HNAU and HPNA groups (HPAU= 87.48 vs HNAU = 75.19 *p* > 0.05; HPAU= 87.48 vs HPNA=85.6 *p* > 0.05). (Controls= 100 vs. HNAU=122.32 *p* > 0.05; Controls=100 vs. HPNA= 167.28 *p* = .0036; Controls=100 vs HPAU= 169.17 *p* = .0063). The permeability/FITC dextran transport in the BBB treated with platelets from HPAU was significantly higher compared to the permeability/FITC dextran transport observed in the BBB treated with platelets from HNAU. (HPAU= 169.17 vs HNAU= 122.32 *p* = .014; HPAU = 169.17 vs HPNA=167.28 *p* > 0.05). Data are expressed as mean percent of controls. Differences were considered significant at *p* ≤ 0.05.

It is well documented that HIV and substance abuse-associated neuro-pathogenesis is marked by a loss of BBB integrity, as previously described [40,41]. However, the role of platelets and thrombocytopenia in this process has not been elucidated yet. To examine the role of platelets as effector cells on the disruption of the Blood Brain Barrier induced by HIV and/or alcohol, the integrity of the BBB model was assessed by TEER measurement in control and treated cultures. Our results, presented in (Figure 1a), show significant BBB disruption when the BBB was treated with platelets from HIV negative alcohol users (HNAU), HIV positive patients (HPNA), and HIV positive alcohol users (HPAU), compared to the controls (CT). Although TEER values were significantly lower in these three groups (HNAU, HPNA, HPAU) compared to the Control Group (CT=100 vs. HNAU=75.19, *p*=0.0057; CT=100 vs. HPNA=85.6, *p*=0.016; CT=100 vs. HPAU=87.48, *p*=0.0106), the BBB treated with platelets from HIV positive alcohol users did not show any significant differences, compared to BBB treated with platelets from HNAU and HPNA groups (HPAU=87.48 vs. HNAU= 75.19, *p*>0.05; HPAU= 87.48 vs. HPNA=85.6, *p*>0.05).

In order to confirm TEER measurement results, para-cellular transport in an *in vitro* BBB model using FITC-dextran as a marker was performed (Figure 1b). The BBB permeability, as demonstrated by FITC-dextran transport, was higher when the BBB was treated with platelets from alcohol users, HIV positive no alcohol users, and HIV positive alcohol users compared to the controls. However, only the BBB treated with platelets from HIV positive patients (HPNA and HIV positive alcohol users (HPAU showed a significant increase in permeability/FITC dextran transport. (Controls= 100 vs. HNAU= 122.32, *p*>0.05; Controls=100 vs. HPNA=167.28, *p*=0.0036; Controls=100 vs. HPAU= 169.17, *p*=0.0063). When further analysis were performed comparing the permeability/FITC dextran transport in the BBB treated with platelets from HPAU, there was a significant difference compared to the permeability/FITC dextran transport observed in the BBB treated with platelets from HNAU. (HPAU=169.17 vs. HNAU= 122.32, *p*=.014; HPAU= 169.17 vs. HPNA=167.28, *p*>0.05).

### Platelets from patients with thrombocytopenia (TCP) decreased the TEER and increased the FITC-dextran transport, affecting BBB integrity

**Figure 2:**
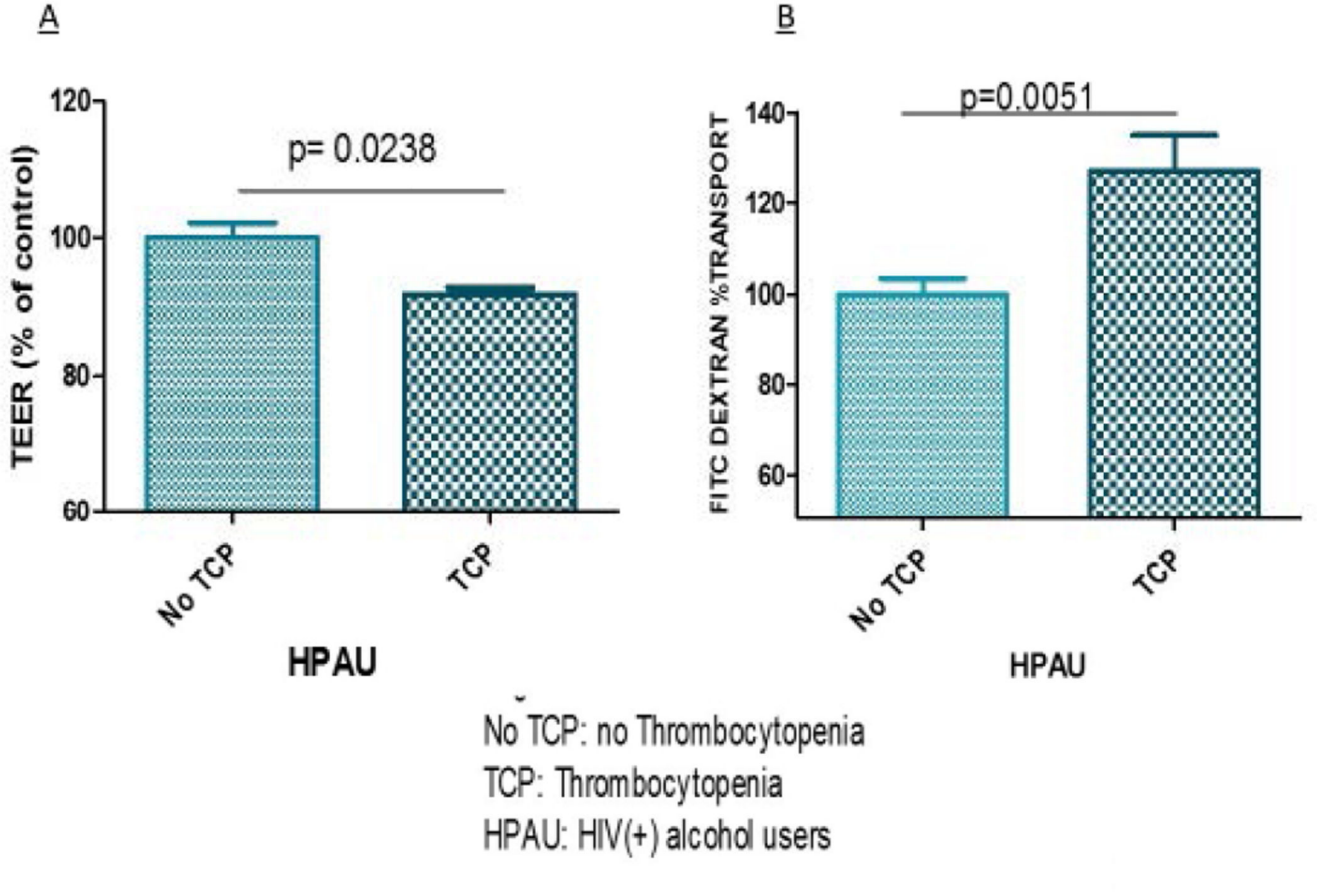
**a, 2b:** Platelets from patients with thrombocytopenia (TCP) decreased the TEER and increased the FITC-dextran transport affecting BBB integrity Platelets from patients with Thrombocytopenia (TCP) decreased the TEER (no TCP =100 vs TCP= 92.0; *p* = .0238) and B) increased the FITC- dextran transport (no TCP= 100.0 vs TCP= 127.0 *p* = .0051) on the context of HIV and Alcohol. Data are expressed as mean percent of controls. Differences were considered significant at *p* ≤ 0.05.

After confirming the involvement of platelets in the drop in TEER values and the increase in FITC dextran transport, we wanted to clarify whether thrombocytopenia was a major cause of the effects seen in the BBB. Since the most robust effects were observed in the BBB that was treated with platelets from HIV positive alcohol users, we proceeded to examine the role of platelets as effector cells on the permeability of the BBB induced by HIV and alcohol. Therefore, the para-cellular permeability using Fluorescein Isothiocyanate (FITC)-dextran under the context of HIV, alcohol and thrombocytopenia was assessed. Platelets from patients with triple co-morbidity (HIV positive, alcohol user and TCP) were studied. Our results indicate that platelets from patients with TCP decreased the TEER (no TCP=100 vs. TCP=92.0, *p*=0.0238) and increased the FITC- dextran transport (no TCP=100.0 vs. TCP=127.0, *p*=0.0051) on the context of HIV and Alcohol (Figure 2a, 2b) respectively.

Analyses indicated that platelets from patients with thrombocytopenia (TCP) decreased the TEER (no TCP=100 vs. TCP=92.0, *p*=0.0238) and increased the FITC- dextran transport (no TCP=100.0 vs. TCP= 127.0, *p*=0.0051), on the context of HIV and Alcohol.

Furthermore, BBB disruption was higher in samples from HIV positive patients and HIV positive alcohol users compared to the controls (controls=85.64 vs. HNND=100, *p*=0.0055; HPAU=87.47 vs. HNND=100, *p*=0.0010; HPND=167.26 vs. HNND=100, *p*=0.0036; HPAU =169.18 vs. HNND =100, *p*=0.0063).

## Discussion

Identifying any factors affecting the integrity of the BBB is at the forefront of research. In this regard, we discovered that platelets from patients with thrombocytopenia affect the intactness (Figure 1a) and permeability (Figure 1b) of the BBB *in vitro*. Although this is the first paper of this nature in the HIV burgeoning literature, similar observations have been reported by others in the context of dengue infection and cerebral malaria [27,28]. Given that the BBB was only exposed to platelets, our findings show the protagonic role of this cell in the disruption of the BBB. These findings are highly relevant, first because it has been confirmed that BBB disruption is one of the mechanisms that underlie HIV associated cognitive impairments [19,20]. Second, findings are highly relevant when considering that up to 4–6 million, out of the 35.3 million people living with HIV, could develop TCP [rate of TCP 15 %], and thus at risk of BBB disruptions [5,17,42]. Such excessive rates should receive more attention, given TCP’s confirmed role on clinical morbidity among PLWH [42–45].

Conceptually, platelets can alter Endothelial Cells (EC) and thereby BBB integrity, by: first, platelet activation can lead to the release of chemokine and inflammatory mediators, such as MIP-1alpha, CCL_5_, MCP-3, CCL17, CXCL1, and CXCL5 [46]. These substances, adjacent to BMECs, can yield clustering of integrin? receptors. Second, HIV that has been shown to directly stimulate the release of platelet sCD40L, leading to up-regulation of MCP-1 and IL-8 within Endothelial Cells [35]. MCP-1, in turn acts as a chemo-attractant, with the consequently release of TNFa, IL-1 and IL-6 [41]. Notably, we have previously demonstrated that thrombocytopenia is associated with an enhanced production of these cytokines in the periphery. Third, evidence has been provided that platelets can participate in immune-mediated cytotoxicity [47,48]. Moreover, platelet-associated GPIIb-IIIa bridging with fibrinogen, can trigger the oxidative burst [49] which, in turn, can induce apoptosis of Endothelial Cells. However, these postulates need to be confirmed in future studies.

Equally relevant, these experiments also show that in the presence of alcohol, platelets deleterious effects are more notorious. The monolayer TEER dramatically decreases, while its permeability increases, suggesting that tight junctions have been compromised (Figure 2a, 2b). This work serves as a breakthrough in the research fields of HIV and alcohol abuse neuro-pathogenesis. Therefore, therapies targeting the platelet system may be an innovative, non-traditional approach for the treatment of HIV and alcohol abused-associated neuro-inflammation.

Nevertheless, our data indicate that alcohol by-itself is sufficient to break the BBB and provide further support to prior reports by Cornford et al. [50]; Elmas et al. [51]; and Haorah et al. [52]. However, our data add to those previous results by indicating that alcohol-induced changes in the platelet system can also contribute to the damages.

When interpreting these findings, it is important to appreciate that this is based on a small sample, and therefore it only represents the first step in this research pathway. Thus additional studies are necessary to build upon these findings. Nonetheless, until further studies emerge, subjects with thrombocytopenia, particularly those with triple comorbidity (TCP+HAU+HIV), should be closely follow-up. Discovering the detailed mechanisms of platelets effects on the BBB will enhance current understanding of HIV associated neurological disorders. More important, it will provide us with additional hints to develop ways to manage PLWH with cognitive disorders.

## Conclusions

In summary, the results show a protagonist role of platelets in the disruption of the BBB and for consequence in mechanisms that underlie cognitive impairments associated with HIV and alcohol consumption. This information can assist in the development of successful treatment approaches for HIV and/or alcohol associated neurological disorders, which, so far, are inexistent.

## Abbreviations

TCP: Thrombocytopenia
HIV: Human Immunodeficiency Virus
ART: Anti-Retroviral Therapy
BBB: Blood Brain Barrier
PADS: The Platelets Mediating Alcohol and HIV Damage Study
PLWH: People Living with HIV
CT: Control Group
HNAU: HIV Negative Alcohol Users
HPAU: HIV Positive Alcohol Users
HPNA: HIV Positive Non-Alcohol Users
TEER: Trans-Endothelial Electrical Resistance
FITC-dextran: Flourescein Isothiocyanate–labeled dextran
